# Editorial Note: Systematic multiscale models to predict the compressive strength of fly ash-based geopolymer concrete at various mixture proportions and curing regimes

**DOI:** 10.1371/journal.pone.0349839

**Published:** 2026-05-21

**Authors:** The PLOS One Editors

The *PLOS One* Editors issue this Editorial Note to inform readers of the following issues that were noted after this article’s [1] publication:

- The article cited as Reference 55 was retracted after [1] was published.
- PLOS has concerns about the article’s adherence to the journal’s first and second publication criteria [2].
